# Epstein-Barr virus DNA loads in the peripheral blood cells predict the survival of locoregionally-advanced nasopharyngeal carcinoma patients

**DOI:** 10.20892/j.issn.2095-3941.2020.0464

**Published:** 2021-08-15

**Authors:** Yongqiao He, Dawei Yang, Ting Zhou, Wenqiong Xue, Jiangbo Zhang, Fangfang Li, Fang Wang, Tongmin Wang, Ziyi Wu, Ying Liao, Meiqi Zheng, Changmi Deng, Danhua Li, Yijing Jia, Leilei Yuan, Wenli Zhang, Weihua Jia

**Affiliations:** 1 Department of Radiation Oncology, Affiliated Cancer Hospital & Institute of Guangzhou Medical University, Guangzhou 510030, China; 2 State Key Laboratory of Oncology in South China, Collaborative Innovation Center for Cancer Medicine, Guangdong Key Laboratory of Nasopharyngeal Carcinoma Diagnosis and Therapy, Sun Yat-sen University Cancer Center, Guangzhou 510030, China; 3 School of Public Health, Sun Yat-sen University, Guangzhou 510030, China; 4 Department of Medical Oncology, Sun Yat-sen University Cancer Center, Guangzhou 510030, China

**Keywords:** Nasopharyngeal carcinoma, Epstein-Barr virus DNA, prognosis, peripheral blood cells, nomogram

## Abstract

**Objective:**

Circulating cell-free Epstein-Barr virus (EBV) DNA has been shown to be a valuable biomarker for population screening and prognostic surveillance for nasopharyngeal carcinoma (NPC). Despite important insights into the biology of persistence, few studies have addressed the clinical significance of cell-based EBV-DNA loads in peripheral blood cells (PBCs).

**Methods:**

A prospective observational cohort study was conducted involving 1,063 newly diagnosed, locoregionally-advanced NPC patients at Sun Yat-sen University Cancer Center from 2005 to 2007. Cox regression analysis was conducted to identify the association of PBC EBV DNA loads to overall survival (OS) and other prognostic outcomes. Prognostic nomograms were developed based on PBC EBV DNA loads to predict survival outcomes for NPC patients.

**Results:**

After a median follow-up of 108 months, patients with higher PBC EBV-DNA loads had significantly worse OS [hazard ratio (HR) of medium, medium-high, and high *vs*. low were 1.50, 1.52, and 1.85 respectively; *P*_trend_ < 0.001]. Similar results were found for progression-free survival and distant metastasis-free survival. The concordance index of the prognostic nomogram for predicting OS in the training set and validation set were 0.70 and 0.66, respectively. Our data showed that the PBC EBV DNA load was an independent and robust survival biomarker, which remained significant even after adjusting for plasma EBV DNA loads in a subset of 205 patients of the cohort (HR: 1.88; *P* = 0.025). Importantly, a combination of PBC EBV DNA load and plasma EBV DNA load improved the predicted OS.

**Conclusions:**

The EBV-DNA load in PBCs may be an independent prognosis marker for NPC patients.

## Introduction

Epstein-Barr virus (EBV) infects more than 95% of adults worldwide and is associated with a diverse range of tumors of both lymphoid and epithelial origins, such as Burkitt lymphoma, Hodgkin’s lymphoma, and nasopharyngeal carcinoma (NPC)^1^. Among these diseases, NPC is characterized by its distinct geographical distribution and is particularly prevalent in southern China, the Arctic, and the middle/northern regions of Africa^2^. The incidence of NPC in southern China is approximately 25–50/100,000 person-years, which is a 20–50-fold increase when compared with Western countries^3–6^. Studies have reported that NPC patients with the same clinical stage receiving similar treatment strategies exhibited distinct clinical outcomes^7^, and that treatment failures were due to high percentages of recurrences and distant metastases^8^. An accurate prognosis in the early treatment stage is therefore necessary to guide precise treatment and follow-up monitoring to reduce the disease burden.

Extensive efforts have been made over the past few decades to discover novel NPC biomarkers for use in clinical practice. Recently, the quantitative assessment of EBV infection has emerged as a promising tool for epidemiological screening, clinical diagnosis, and surveillance of recurrence. The virus is able to infect both B cells and epithelial cells, and shuttle between these 2 cells, facilitating its persistence and transmission in humans^9–11^. Samples from different blood compartments [e.g., serum/plasma, peripheral blood mononuclear cells (PBMCs), and whole blood] are therefore often used to monitor EBV infection in clinical or epidemiological studies of NPC^12,13^. The measurement of viral DNA from different blood compartments, however, yields very different information in certain situations due to the complexity of EBV kinetics, which affects the distribution, persistence, and interchange of EBV among plasma and PBMCs^14^. For example, the plasma cell-free EBV DNA (cfEBV DNA), which is mainly derived from tumor cells, appears to correlate closely with the presence of residual tumors. Thus, measuring plasma cfEBV DNA provides an almost real-time readout of the tumor burden, and is useful for monitoring the recurrence, prognostication, treatment response prediction, and disease surveillance of NPC^8,15–22^. However, the EBV DNA in PBMCs, typically harbored within latently infected B lymphocytes^9,23,24^, does not appear promising for either tumor diagnoses or therapeutic effect evaluations in NPC clinical practice due to its lower detection^25^. Nevertheless, measuring EBV copy number in PBMCs has provided important insights into the biology of persistence and the role of the resting memory B cells. It might serve as a general marker of immune function, which may have prognostic significance among patients regardless of tumor EBV status^26^. Indeed, a previous study reported that 4 of 9 patients with detectable EBV DNA in peripheral blood cells (PBCs) had tumor relapses, whereas none of the 4 patients without detectable EBV DNA in PBCs developed a tumor relapse, suggesting that EBV DNA in PBCs could be a prognostic biomarker for NPC^27^.

However, few studies have reported the clinical application of EBV DNA loads in PBCs, particularly its prognostic value in NPC, so we conducted the first prospective observational cohort study with a relatively large sample size and longer follow-up. In this study, we aimed to determine whether EBV DNA loads in PBCs could be used as a prognostic marker. If so, we aimed to determine if it was correlated with current prognostic markers such as plasma EBV DNA loads, and if the combination of EBV DNA loads in both plasma and PBCs could improve the predictive value for prognoses.

## Materials and methods

### Study population

The present hospital-based cohort study was conducted on the basis of the EPI-NPC-2005 project during October 2005 and October 2007, which was designed to assess the interaction among EBV, environmental factors, and genetic determinants in the pathogenesis and progression of NPC. Briefly, NPC patients were recruited from the Sun Yat-sen University Cancer Center (SYSUCC; Guangzhou, China), according to the following criteria: 1) newly-diagnosed NPC patients without other malignancy histories; 2) pathologically confirmed as World Health Organization type II and III NPC patients; 3) under 80 years of age; and 4) residing in the Guangdong Province for at least 5 years. This study further included patients from the EPI-NPC-2005 according to the following criteria: 1) diagnosed as locoregionally-advanced NPC patients with T_x_N_2-3_M_0_ or T_2-4_N_0-3_M_0_ (6th AJCC/UICC TNM staging systems); 2) had been treated with radiotherapy; and 3) having blood samples collected prior to radiotherapy or within 2 weeks after the start of radiotherapy. Finally, a total of 1,063 eligible patients were included. The flow chart of patient inclusion is shown in **Supplementary Figure S1**. Written informed consent was obtained from each participant at enrollment. The study was approved by the Ethics Committee of SYSUCC (YB2005001).

### Data collection

Personal information was collected using a structured questionnaire through face-to-face interviews by trained staff. The collected information mainly included demographics (age, gender, and levels of education), lifestyle habits (smoking behavior, alcohol drinking, etc.) and disease history (hypertension, diabetes, and heart disease). Medical information included clinical stage, T and N stage, radiotherapy technology [2-dimensional radiotherapy (2D-RT), 3-dimensional conformal radiotherapy (3D-CRT), and intensity modulated radiation therapy (IMRT)], chemotherapy (induced chemotherapy, concurrent chemotherapy, and adjuvant chemotherapy), and other information, which was retrieved from the hospital information system of SYSUCC. The raw data for this study have been uploaded to the Research Data Deposit with a number of RDDA2020001667.

### Blood processing and DNA extraction

Peripheral blood (4 mL) was collected from each patient in an EDTA tube. The whole blood was centrifuged at 3,500 × *g* for 10 min, and the supernatant was removed. Cell pellets were transferred into 1.5 mL centrifuge tubes and centrifuged at 850 × *g* for 5 min, and then washed 3 times with phosphate-buffered saline for PBC DNA extractions. The DNA of PBCs was extracted using a QIAamp DNA Blood MiniKit (Qiagen, Hilden, Germany). The final volume was 50 μL.

### The real-time quantitative polymerase chain reaction

The EBV DNA loads were measured using a real-time quantitative PCR assay targeting the *BamHI-W* region of the EBV genome. The detailed method has been reported in our previous study^28^. The *BamHI-W* system consisted of the amplification primers W-44F: (5′-CCC AAC ACT CCA CCA CACC-3′) and W-119R: (5′-TCT TAG GAG CTG TCC GAG GG-3′) and the dual-labeled fluorescent probe W-67T: [5′-(FAM) CAC ACA CTA CAC ACA CCC ACC CGT CTC (TAMRA)-3′]. Real-time quantitative PCR for the β-globin gene consisted of the amplification primers β-globin-354F: (5′-GTG CAC CTG ACT CCT GAG GAG-3′) and β-globin-455R: (5′-CCT TGA TAC CAA CCT GCC CAG-3′) and the dual-labeled fluorescent probe β-globin-402T: [5′-(FAM) AAG GTG AAC GTG GAT GAA GTT GGT GG (TAMRA)-3′]^29^. The fluorescent probes and PCR primers were custom-synthesized by Life Technologies (Carlsbad, CA, USA). Standard samples that contained the *BamHI-W* gene or β-globin gene were constructed from the pMD19-T simple vector (TaKaRa, Shiga, Japan), termed pMD19-T-BamHI-W and pMD19-T-β-globin, respectively. The standard curves were generated with serially diluted standard samples at 10^3^,10^4^, 10^5^, 10^6^, and 10^7^ copies per 2 μL.

Each PCR reaction was conducted in a total volume of 8 μL, which contained 2× MasterMix (Roche, Basel, Switzerland), 4 μL of 100 nM of each of the amplification primers, 100 nM of the corresponding fluorescent probe, and 2 μL of DNA templates. Duplicate amplification reactions were performed in 384-well microplates using a Roche LightCycler 480 amplifier. Standard curves were run in parallel and in duplicate with each assay. Thermal cycling was initiated at 10 min at 95 °C for the first denaturation, then followed by 40 cycles of 95 °C for 15 s, 60 °C for 1 min, and 72 °C for 45 s. The β-globin gene quantitative PCR results were used to normalize the quantity of EBV DNA. The EBV DNA load in PBCs was expressed as 10^6^-fold of the ratio of EBV DNA copies to the β-globin copies in the same tested samples^29,30^. Multiple blanks were used as negative controls. The mean quantity of each duplicate was used for further calculations for the concentrations of EBV DNA. The equation to calculate EBV copies per β-globin was:

$$\begin{array}{l} \text{PBC EBV copies}/10^{6}\text{globin}=(\text{EBV mean quantity}/\\ \beta -\text{globin mean quantity})\times 10^{6}. \end{array}$$

### Follow-up and outcomes

Patients were followed-up mainly by telephone interviews, retrieving the HIS medical records, and accessing death registration data from the public security bureau. The follow-up period ended in September 2019. The main endpoints of this study were death, disease progression, distant metastasis, and recurrence. Distant metastasis and recurrence were defined as the appearance of a newly detected local/regional recurrence or distant metastasis, and were confirmed by nasopharyngeal biopsy or 2 kinds of imaging diagnoses. Overall survival (OS) was determined as the date of diagnosis to the date of death or to the date of the last follow-up visit. Progression-free survival (PFS) was defined as the time from the date of diagnosis to the first day of local recurrence or distant metastasis or to the date of the last follow-up visit. Distant metastasis-free survival (DMFS) was calculated from the date of diagnosis to the date of distant metastasis or the date of the last follow-up visit. Recurrence-free survival (RFS) was calculated from the date of diagnosis to the date of local and/or regional recurrence or the date of the last follow-up visit.

### Statistical analysis

Life table estimation was performed using the method of Kaplan-Meier. Univariate comparison of survival curves was performed using the log-rank test. Univariate Cox regression was performed to screen for significant variables. The multivariate Cox proportional hazards model was used to estimate hazard ratios and 95% confidence intervals. Adjusted variables in the model included age, gender, smoking status, clinical stage, radiotherapy technology, chemotherapy, and other factors that were significant in univariate Cox regression. To properly display the dose-response relationship between EBV DNA loads in PBCs and the prognoses of NPC patients, we defined cut-off values based on the quartiles of patients with detectable PBC EBV DNA. We classified these patients with 0 copies/10^6^ globin and the first quartile of PBCs EBV DNA as the low group (≤ 392 copies/10^6^ globin), second quartile as the medium group (> 392 copies/10^6^ globin and ≤ 581 copies/10^6^ globin), third quartile as the medium-high group (> 581 copies/10^6^ globin and ≤ 918 copies/10^6^ globin), and the top quartile as the high group (> 918 copies/10^6^ globin). Using subgroup analysis by clinical stage, the patients were categorized into low or high groups (≤ 392 copies/10^6^ globin and > 392 copies/10^6^ globin, respectively) because of the limited sample size in each subgroup. All statistical tests were 2-sided, and *P* < 0.05 was considered statistically significant. Analyses were performed using R 3.6.1 software (The R Project for Statistical Computing, Vienna, Austria).

The nomograms were developed to predict the status of OS and PFS based on the results of univariate Cox regression analyses. Two-thirds of 1,063 patients were randomly assigned to the training set and one-third to the validation set. In addition, the 2 markers of lactate dehydrogenase (LDH) and neutrophil to lymphocyte ratio (NLR) that were associated with prognosis of NPC patients in previous publications were also included in the nomogram^31,32^. The performance of the nomogram was evaluated by the concordance index (C-index) in the training and validation sets. A calibration curve of the nomogram was used to estimate agreement between the predictions and observations regarding the probabilities of 3-, 5-, and 10-year survivals.

To study the relationship between plasma EBV DNA and PBC EBV DNA loads, 205 of 1,063 samples with available pretreatment plasma EBV DNA loads were retrieved from the HIS of SYSUCC. The correlations were tested using Spearman’s rank correlation. We classified plasma EBV DNA and PBC EBV DNA loads into the high and low categories, respectively. The optimal threshold of plasma EBV DNA loads was 128,000 copies/mL based on receiver operating characteristic curve analyses and the cut-off value of PBC EBV DNA loads was consistent with that of the subgroup analyses (392 copies/10^6^ globin). We further combined the 2 markers and classified the population into a low (both markers were low), medium (1 of the 2 markers was low), and high (both markers were high) groups. The C-index was calculated to evaluate the prognostic effect of the 2 markers, alone and in combination.

## Results

### Patient characteristics and follow-up

The baseline characteristics of the 1,063 locoregionally-advanced NPC patients are summarized in **Supplementary Table S1**. The median age of the population was 45 years [interquartile range (IQR): 38–54 years], with a male-to-female ratio of 2.7 (775 males and 288 females). Based on the 6th edition of AJCC/UICC staging system, 178 patients (16.7%) were in stage II, 601 patients (56.5%) in stage III, and 284 patients (26.7%) in stage IV. The detection percentage of the PBC EBV DNA loads was 38.9% (413/1,063) in all patients. The median PBC EBV DNA loads were 0 copies/10^6^ globin (IQR = 0–0), 469 copies/10^6^ globin (IQR = 425–521), 714 copies/10^6^ globin (IQR = 641–793), and 1,428 copies/10^6^ globin (IQR = 1,103–2,342) in the low (752 patients), medium (104 patients), medium-high (103 patients), and high (104 patients) groups, respectively (**Supplementary Figure S2**).

The median follow-up time was 107.93 months (range: 2.47–163.53 months), and 431 (40.5%) patients died, 466 (43.8%) experienced disease progression, 196 (18.4%) developed distant metastasis, and 137 (12.9%) developed local and/or regional recurrences during the follow-up period.

### Survival status and factors associated with the OS, PFS, DMFS, and RFS of NPC patients

The overall survival rates of NPC patients at 3-, 5-, and 10-years were 89%, 79%, and 61%, respectively. The PFS rates at 3-, 5-, and 10-years were 80%, 72%, and 58%, respectively. The DMFS rates at 3-, 5-, and 10-years were 87%, 83%, and 80%, respectively, and the RFS rates at 3-, 5-, and 10-years were 93%, 90%, and 85%, respectively.

Univariate analyses indicated that age, gender, education levels, smoking status, clinical stage, T stage, N stage, radiotherapy technology (2D-RT, 3D-CRT, and IMRT), induced chemotherapy, and concurrent chemotherapy were significantly associated with the OS and PFS. Of these parameters, age, smoking status, clinical stage, T stage, N stage, and induced chemotherapy were risk factors, while higher education levels, female, using radiotherapy technology of intensity-modulated radiation therapy, and concurrent chemotherapy were protective factors. In addition, gender, smoking status, clinical stage, and concurrent chemotherapy were correlated with both the DMFS and RFS. The T stage and N stage were also correlated with the DMFS (**Supplementary Table S2**).

Multivariate analyses showed that age, gender, clinical stage, and concurrent chemotherapy were associated with the OS and PFS. High levels of education were a protective factor for OS. Gender, clinical stage, and concurrent chemotherapy were independent factors for the DMFS. Only clinical stage and concurrent chemotherapy were significant for RFS when using multivariate analysis (**Supplementary Table S3**).

### The prognostic value of EBV DNA loads in PBCs

We found positive associations between PBC EBV-DNA loads and clinical stage characteristics in NPC patients. A significant association was found between high levels of PBC EBV-DNA and overall clinical stage progressing [III *vs*. II: odds ratio (OR): 1.71, 95% confidence interval (CI): 1.05–2.79; *P* = 0.032; IV *vs*. II: OR: 2.45; 95% CI: 1.41–4.24; *P* < 0.001; *P*_trend_ < 0.001). Patients with a body mass index (BMI*)* more than 24 had significantly lower PBC EBV DNA loads than those with a normal BMI (OR: 0.70; 95% CI: 0.50–0.97; *P* = 0.033). The results are summarized in **Supplementary Table S4**.

**Figure 1:**
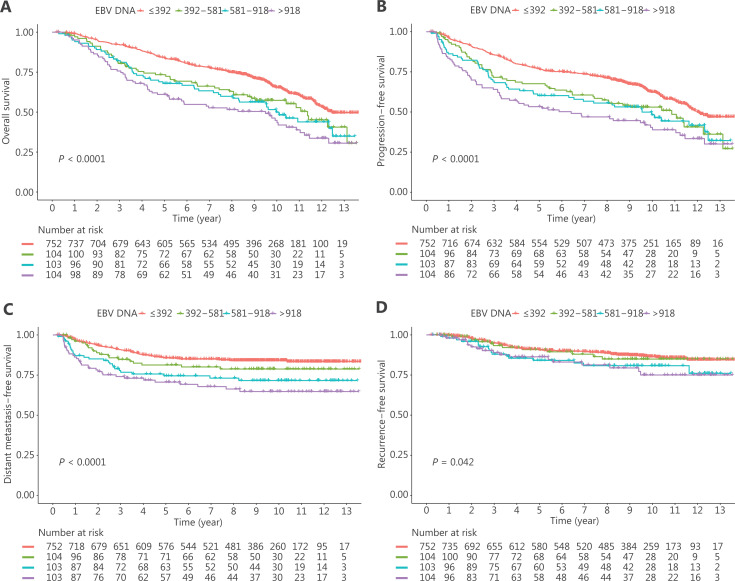
Kaplan-Meier survival curves of Epstein-Barr virus DNA in peripheral blood cells for (A) overall survival, (B) progression-free survival, (C) distant metastasis-free survival, and (D) recurrence-free survival in all patients.

The overall survival at 5 years of patients in the low, medium, medium-high, and high groups of EBV DNA loads were 83.9%, 73.4%, 67.9%, and 61.1%, respectively, which showed a gradually decreased EBV DNA load with a stepwise rise. Similar results at 5 years were also observed for the PFS (76.7%, 67.6%, 60.2%, and 53.3% in the low, medium, medium-high, and high groups, respectively), DMFS (85.7%, 81.3%, 74.5%, and 70.6% for the low, medium, medium-high, and high groups, respectively) and RFS (90.7%, 90.8%, 84.1%, and 86.3% for the low, medium, medium-high, and high groups, respectively). The survival curves are showed in **Figure 1**.

Multivariate Cox regression analyses identified EBV DNA loads in PBCs as an independent prognostic factor for the OS. Compared with the low group of EBV DNA loads in PBCs, the HRs in the medium, medium-high, and high EBV DNA loads were 1.50 (95% CI: 1.10–2.05; *P* = 0.010), 1.52 (95% CI: 1.12–2.07; *P* = 0.007), and 1.85 (95% CI: 1.40–2.46; *P* < 0.001), respectively, *P*_trend_ < 0.001. Similar positive associations were also found for the PFS [medium, medium-high, high *vs*. low: HR: 1.50 (*P* = 0.007); 1.46 (*P* = 0.013); 1.85 (*P* < 0.001), respectively, *P*_trend_ < 0.001] and the DMFS [medium, medium-high, high *vs*. low: HR: 1.31 (*P* = 0.272); 1.76 (*P* = 0.009); 2.37 (*P* < 0.001), respectively;* P*_trend_ < 0.001]. Only the high PBC EBV DNA group had a significantly worse RFS relative to the low PBC EBV DNA groups (HR: 1.70; *P* = 0.047), but there was a significantly linear trend with a greater probability of recurrence with increasing EBV DNA loads in PBCs (*P*_trend_ = 0.023) (**Table 1 and Supplementary Table S3**).

**Table 1:** Multivariate Cox regression analysis of peripheral blood cell Epstein-Barr virus DNA loads in all patients

Characteristics	Overall survival^a^			Progression-free survival^a^			Distant metastasis-free survival^b^			Recurrence-free survival^b^		
	*n*./*N*.	HR (95%CI)	*P*	*n*./*N*.	HR (95%CI)	*P*	*n*./*N*.	HR (95%CI)	*P*	*n*./*N*.	HR (95%CI)	*P*
≤ 392 copies/10^6^ globin	269/752	Reference	–	294/752	Reference	–	115/752	Reference	–	90/752	Reference	–
392–581 copies/10^6^ globin	49/104	1.50 (1.10–2.05)	0.010	54/104	1.50 (1.12–2.01)	0.007	20/104	1.31 (0.81–2.14)	0.272	12/104	1.05 (0.56–1.96)	0.889
581–918 copies/10^6^ globin	51/103	1.52 (1.12–2.07)	0.007	54/103	1.46 (1.08–1.96)	0.013	27/103	1.76 (1.15–2.69)	0.009	17/103	1.61 (0.95–2.72)	0.077
> 918 copies/10^6^ globin	62/104	1.85 (1.40–2.46)	< 0.001	64/104	1.85 (1.41–2.44)	< 0.001	34/104	2.37 (1.61–3.51)	< 0.001	18/104	1.70 (1.01–2.87)	0.047
*P*_trend_			< 0.001			< 0.001			< 0.001			0.023

*n*: the number of events; *N*: the total number of patients in each group. a: Adjusted for age, gender, smoking status, T stage, N stage, radiotherapy technology, chemotherapy (induced chemotherapy, concurrent chemotherapy, and adjuvant chemotherapy), and education levels. b: Adjusted for age, gender, smoking status, T stage, N stage, radiotherapy technology, and chemotherapy (induced chemotherapy, concurrent chemotherapy, and adjuvant chemotherapy).

To further investigate the prognostic value of EBV DNA loads in PBCs, we performed subgroup analyses based on clinical stages. Multivariate Cox regression analyses in the subgroup of clinical stage II indicated that PBC EBV DNA was significantly correlated with worse outcomes of the OS (HR: 2.72; *P* = 0.008), PFS (HR: 3.38; *P* < 0.001), DMFS (HR: 4.96; *P* = 0.003), and RFS (HR: 3.31; *P* = 0.039). The PBC EBV DNA was significantly associated with the OS in clinical stage subgroup III (*P* = 0.025). In the clinical stage IV group, which had the worse prognosis, patients with a higher EBV DNA load in the PBCs had a worse OS (HR: 1.92; *P* < 0.001), PFS (HR: 1.93; *P* < 0.001), DMFS (HR: 2.07; *P* = 0.002), and RFS (HR: 2.15; *P* = 0.015) (**Table 2**, **Supplementary Figure S3, and Supplementary Table S5–S7**).

**Table 2:** Multivariate Cox regression analysis of peripheral blood cell Epstein-Barr virus DNA loads in subgroups of clinical stages

Characteristics	Overall survival^a^			Progression-free survival^a^			Distant metastasis-free survival^b^			Recurrence-free survival^b^		
	*n*./*N*.	HR (95%CI)	*P*	*n*./*N*.	HR (95%CI)	*P*	*n*./*N*.	HR (95%CI)	*P*	*n*./*N*.	HR (95%CI)	*P*
Subgroup of clinical stage II												
≤ 392 copies/10^6^ globin	27/148	Reference	–	29/148	Reference	–	9/148	Reference	–	11/148	Reference	–
> 392 copies/10^6^ globin	11/30	2.72 (1.29–5.73)	0.008	14/30	3.38 (1.72–6.65)	< 0.001	7/30	4.96 (1.71–14.34)	0.003	5/30	3.31 (1.06–10.31)	0.039
Subgroup of Clinical stage III												
≤ 392 copies/10^6^ globin	153/428	Reference	–	169/428	Reference	–	68/428	Reference	–	56/428	Reference	–
> 392 copies/10^6^ globin	76/173	1.38 (1.04–1.82)	0.025	80/173	1.27 (0.97–1.67)	0.081	36/173	1.42 (0.94–2.14)	0.093	20/173	0.97 (0.58–1.63)	0.919
Subgroup of Clinical stage IV												
≤ 392 copies/10^6^ globin	89/176	Reference	–	96/176	Reference	–	38/176	Reference	–	23/176	Reference	–
> 392 copies/10^6^ globin	75/108	1.92 (1.40–2.63)	< 0.001	78/108	1.93 (1.41–2.63)	< 0.001	38/108	2.07 (1.30–3.29)	0.002	22/108	2.15 (1.16–3.98)	0.015

*n*: the number of events; *N*: the total number of patients in each group. a: Adjusted for age, gender, smoking status, T stage, N stage, radiotherapy technology, chemotherapy (induced chemotherapy, concurrent chemotherapy, and adjuvant chemotherapy), and education levels. b: Adjusted for age, gender, smoking status, T stage, N stage, radiotherapy technology, and chemotherapy (induced chemotherapy, concurrent chemotherapy, and adjuvant chemotherapy).

We conducted subgroup analyses using different treatment stages to further determine whether the PBC EBV DNA loads were correlated with NPC survival in different treatment stages. In our study, 630 patients (59.3%) were recruited before any treatment, 247 (23.2%) were recruited during induced chemotherapy, and 186 (17.5%) were recruited within 2 weeks after the start of radiotherapy. We conducted multivariate Cox regression analyses according to different treatment stage subgroups and found that PBC EBV DNA loads were significantly associated with the OS and PFS within each subgroup of the treatment stages (**Supplementary Figure S4**).

### Nomogram development using the EBV DNA loads in PBCs

**Figure 2:**
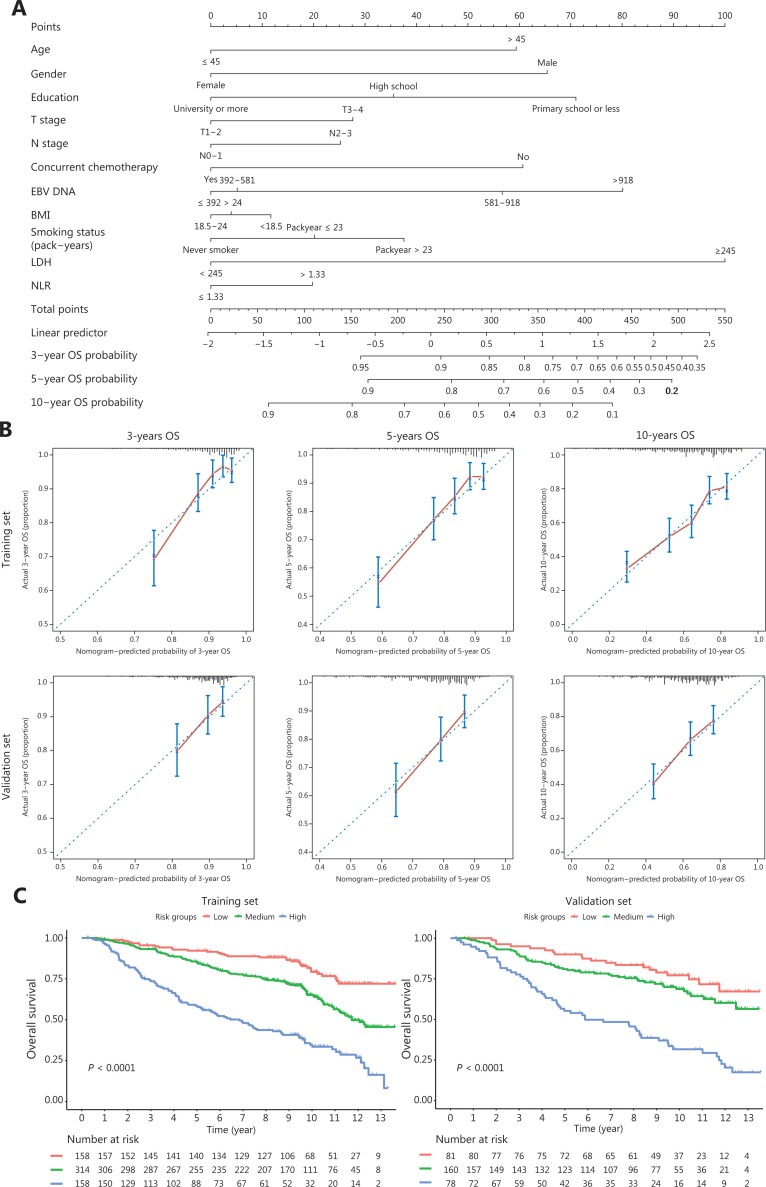
Nomogram (A), including age, gender, education level, T stage, N stage, concurrent chemotherapy, body mass index, smoking status, serum lactate dehydrogenase levels, and neutrophil to lymphocyte ratio for 3-, 5-, and 10-year overall survival (OS) in patients with nasopharyngeal carcinoma. The calibration curve (B) of the nomogram for predicting OS. The Kaplan-Meier curves (C) of different risk groups in the training and validation sets according to the score system for the OS.

To investigate the clinical prognostic value of EBV DNA loads in PBCs, we constructed the prognostic nomograms using the training set to predict the 3-, 5-, and 10-year OS using the variables of EBV DNA loads in PBCs, age, gender, education level, T stage, N stage, concurrent chemotherapy, BMI, smoking status, LDH, and NLR (**Figure 2A**). The calibration plot for the probability of 3-, 5-, and 10-year OS revealed favorable agreement between predictions by the nomogram and actual observations in both the training and validation sets (**Figure 2B**). The C-indices for the OS in the training and validation sets were 0.70 (95% CI: 0.66–0.73) and 0.66 (95% CI: 0.61–0.71), respectively, indicating that the nomogram with PBC EBV DNA loads had good prognostic stratification. Moreover, patients in the training set were categorized into 3 risk groups at the 25th and 75th percentiles of the total score distribution of the training set: low risk group (total score: 0–175), medium risk group (total score: 176–290), and high risk group (total score ≥ 291), and each group showed a distinct prognosis (**Supplementary Table S8**). In the validation set, the 3-, 5-, and 10-year OS rates were 94%, 90%, and 77%, respectively, in the low risk group, 89%, 80%, and 68% in the medium risk group, respectively, and 76%, 55%, and 31% in the high risk group, respectively. **Figure 2C** shows the Kaplan-Meier survival curves of different risk groups for the OS in the training and validation sets. In addition, similar results for the PFS were also found (**Supplementary Table S8 and Supplementary Figure S5**).

### The relationship between plasma EBV DNA and PBC EBV DNA loads

**Figure 3:**
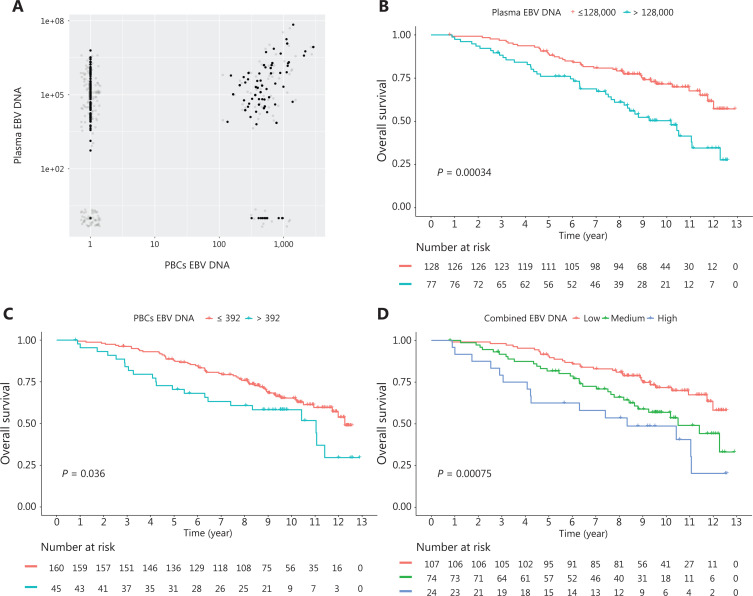
A scatter plot (A) indicating the correlation between plasma Epstein-Barr virus (EBV) DNA and peripheral blood cell (PBC) EBV DNA. Grep points are the jittered points. Kaplan-Meier survival curves of overall survival. (B) plasma EBV DNA alone, (C) PBC EBV DNA alone, (D) The combination of plasma EBV DNA and PBC EBV DNA.

To determine whether PBC EBV DNA loads were independent of plasma EBV DNA loads, a correlation analysis was conducted of 205 patients who had available data on both of the 2 markers in our cohort. Notably, we observed a weak correlation between plasma EBV DNA and PBC EBV DNA loads (**Figure 3A**; *R*^2^: 0.32; *P* < 0.001). Multivariate Cox regression including the 2 EBV DNA loads and other adjusting factors showed PBC EBV DNA loads were independent of plasma EBV DNA loads for the OS (HR: 1.88; *P* = 0.025; **Supplementary Table S9**). We further compared the predictive effects of the 2 markers alone and in combination. The C-index of PBC EBV DNA loads, plasma EBV DNA loads, and combination of the 2 markers were 0.56, 0.59, and 0.61, respectively, indicating that the addition of PBC EBV DNA loads improved the predictive accuracy of plasma EBV DNA loads. Kaplan-Meier curve analyses indicated that the combination of 2 markers further identified patients with worse prognoses than either of the 2 markers alone (**Figure 3B–3D**).

## Discussion

In the present study, we observed a quantitative correlation between PBC EBV DNA loads and the prognoses of locoregionally-advanced NPC patients. Importantly, nomograms with PBC EBV DNA loads and other relative factors showed an improved accuracy in the prediction for OS and PFS, which could be utilized as clinical tools to further distinguish subgroups of NPC patients with different prognoses.

Previous studies have reported that high levels of EBV DNA in PBMCs were associated with increased risks of developing EBV-associated diseases, such as AIDS-related systemic B lymphoma and post-transplant lymphoproliferative disease^33–35^. Lin et al.^36^ detected the presence of EBV DNA in PBCs by nested PCR among 124 NPC patients, and qualitatively showed that EBV DNA positive patients had an inferior OS and DMFS, when compared with patients with a negative EBV DNA in their PBCs. In the present study, we used a more sensitive method to detect EBV DNA loads in PBCs, to establish a quantitative relationship between EBV DNA loads and the survival outcomes of NPC patients, using our NPC cohort with larger sample sizes and a longer follow-up. The survival data of patients (OS, PFS, DMFS, and RFS) were significantly decreased with increased EBV DNA loads in their PBCs, and these associations remained consistent in each subgroup of patients with clinical stages II, III, and IV. Moreover, our nomogram with PBC EBV DNA loads and other risk factors showed good predictive performance in identifying subgroups of patients with poor survivals. Using our scoring system, physicians could predict individual survival, which would improve treatment and health care. The impact of EBV-infected PBCs on the prognoses of NPC patients may be related to the ability of the virus to counteract and evade host immunity by modulating cellular signaling pathways, blocking antiviral cytokines, impairing the antigen-presenting HLA I or the HLA II pathway, and switching-off immunodominant viral antigen expression as well as regulation of immune-inhibitory biomolecules^37^. The detailed molecular mechanisms underlying the role of EBV infected PBCs in NPC prognosis therefore need further investigation.

An early study using a prognostic model for NPC indicated that including plasma EBV DNA loads in the prognostic prediction significantly increased the predictive accuracy and discriminative ability^16,38^. Because plasma EBV DNA is mainly released by tumor cells, it has been useful for the diagnosis, occurrence, development, and prognoses of EBV-associated diseases, including NPC^39^. Significant progress was recently made by Chan et al.^15^, who used plasma EBV DNA in screening for early NPC, with the sensitivity and specificity as high as 97.1% and 98.6%, respectively. Moreover, Lv et al.^18^ successfully regrouped NPC patients into different prognostic phenotypes by monitoring cfEBV DNA longitudinally, and Kanakry et al.^40^ also showed that plasma EBV DNA had prognostic utility for Hodgkin lymphoma patients.

To further explore the impact of plasma EBV DNA and PBC EBV DNA loads on the accuracy of NPC prognostic predictions, we selected 205 patients with pretreatment plasma EBV DNA loads among all patients. Our analyses showed a weak correlation between the 2 markers, showing that PBC EBV DNA loads were independent of plasma EBV DNA loads. The significant association between PBC EBV DNA loads and the prognoses of NPC patients was confirmed after adjusting the plasma EBV DNA loads and other parameters. Shao et al.^25^ reported that plasma EBV DNA loads were not correlated with PBC EBV DNA loads in NPC patients before treatment, and Gandhi et al.^41^ also reported that EBV DNA loads of EBV-positive Hodgkin’s lymphoma patients in matched plasma/PBMC samples were not correlated. Consistent with these previous studies, we hypothesized that cell-based EBV DNA in PBCs may be slightly associated with plasma EBV DNA loads, and that PBC EBV DNA loads could be an independent prognostic factor relative to plasma EBV DNA loads.

Previous studies focused on which blood compartments for detection and quantification of EBV DNA could better reflect the diagnosis and prognosis of EBV-associated disease^26,42,43^. However, EBV DNA loads from different blood compartments may have different biological significances. For example EBV DNA loads in the plasma reflect tumor burden in NPC patients^20^, and EBV DNA loads in PBMCs may be more related to body immunity in post-transplant lymphoproliferative disorders^44^. The combined use of EBV DNA from different blood compartments may be able to more comprehensively reflect the status of the disease. In our study, the combination of PBC EBV DNA and pretreatment plasma EBV DNA loads further differentiated between higher risk groups and showed better performance in NPC prediction than plasma EBV DNA loads alone.

Our study had some limitations. First, because 2-dimensional radiation therapy was the standard radiation technique during enrollment, only a small percentage of patients underwent radiotherapy using intensity-modulated radiation therapy. Second, although the relationship between PBC EBV DNA loads and prognosis was significant, based on our cohort with a large sample size and longer follow-up times, we suggest the future use of multi-center studies with replication results to better verify these associations. In addition, our available data and samples for simultaneous EBV DNA load detection for both plasma and PBCs were relatively limited, and the conclusions from these samples needs to be confirmed, using larger sample sizes and other well-designed studies.

## Conclusions

In summary, our study showed that EBV DNA loads in PBCs may be an important independent prognostic marker for locoregionally-advanced NPC patients relative to plasma EBV DNA loads. The proposed nomogram with PBC EBV DNA loads in this study provided good discrimination in predicting OS and disease progression.
