# Supplementary material for: Epstein-Barr virus DNA loads in the peripheral blood cells predict the survival of locoregionally-advanced nasopharyngeal carcinoma patients
Source: Cancer Biol Med. 2021 Aug 15;18(3):888–99. doi: 10.20892/j.issn.2095-3941.2020.0464 (PMC8330545; doi:10.20892/j.issn.2095-3941.2020.0464)
Supplement: Supplementary file 1 [file cbm-18-888-s001.pdf]

Supplementary materials

Table S1 Patient demographics and clinical characteristics

Characteristics	No. of patients	%
Age (years)		
≤ 45	565	53.2
> 45	498	46.8
Gender		
Male	775	72.9
Female	288	27.1
BMI		
18.5–24.0	576	59.0
> 24.0	301	30.8
< 18.5	100	10.2
Education		
Primary school or less	244	23.0
High school	663	62.4
University or more	156	14.7
Smoking status (pack-years)		
Never smoker	493	46.6
≤ 23	288	27.2
> 23	277	26.2
Alcohol drinking		
Nondrinker	628	59.1
≤ 1 drink per day	251	23.6
> 1 drink per day	184	17.3
Clinical stage		
II	178	16.7
III	601	56.5
IV	284	26.7
T stage		
T_{1-2}	297	27.9
T_{3-4}	766	72.1
N stage		
N_{0-1}	670	63.0
N_{2-3}	393	37.0
Radiotherapy technology		
2D-RT	869	82.3
3D-CRT	14	1.3
IMRT	173	16.4
Induced chemotherapy		
No	548	51.6
Yes	515	48.4
Concurrent chemotherapy		
No	621	58.4
Yes	442	41.6
Adjuvant chemotherapy		
No	1,017	95.7
Yes	46	4.3
Groups by PBC EBV DNA loads (copies/10^6 globin)		
0	650	61.1
(0–392]	102	9.6
(392–581]	104	9.8
(581–918]	103	9.7
> 918	104	9.8
History of hypertension		
No	994	93.5
Yes	69	6.5
History of diabetes		
No	1,031	97.0
Yes	32	3.0
History of heart disease		
No	1,055	99.2
Yes	8	0.8
Overall survival status		
No	632	59.5
Yes	431	40.5
Progress-free survival status		
No	597	56.2
Yes	466	43.8

Table S1 Continued

Characteristics	No. of patients	%
Distant metastasis-free survival status		
No	867	81.6
Yes	196	18.4
Recurrence-free survival status		
No	926	87.1
Yes	137	12.9

Table S2 Univariate Cox regression analysis of all patients

Characteristics	Overall survival			Progression-free survival			Distant metastasis-free survival			Recurrence-free survival		
	n./N.	HR (95%CI)	*P*	*n./N.*	HR (95%CI)	*P*	*n./N.*	HR (95%CI)	*P*	*n./N.*	HR (95%CI)	*P*
Age (years)												
≤ 45	176/565	Reference	–	201/565	Reference	–	95/565	Reference	–	72/565	Reference	–
> 45	255/498	1.86 (1.53–2.25)	< 0.001	265/498	1.64 (1.36–1.97)	< 0.001	101/498	1.26 (0.95–1.67)	0.108	65/498	1.09 (0.78–1.53)	0.612
Gender												
Male	347/775	Reference	–	371/775	Reference	–	164/775	Reference	–	109/775	Reference	–
Female	84/288	0.56 (0.44–0.71)	< 0.001	95/288	0.59 (0.47–0.74)	< 0.001	32/288	0.49 (0.33–0.71)	< 0.001	28/288	0.61 (0.40–0.93)	0.021
BMI												
18.5–24.0	235/576	Reference	–	258/576	Reference	–	111/576	Reference	–	73/576	Reference	–
> 24.0	116/301	0.88 (0.70–1.10)	0.259	122/301	0.84 (0.68–1.05)	0.120	48/301	0.79 (0.56–1.11)	0.174	38/301	0.94 (0.64–1.39)	0.759
< 18.5	39/100	0.90 (0.64–1.26)	0.545	43/100	0.90 (0.65–1.24)	0.503	18/100	0.92 (0.56–1.51)	0.742	12/100	0.91 (0.49–1.68)	0.762
Education												
Primary school or less	119/244	Reference	–	125/244	Reference	–	48/244	Reference	–	31/244	Reference	–
High school	268/663	0.77 (0.62–0.95)	0.017	288/663	0.80 (0.65–0.99)	0.041	127/663	0.96 (0.69–1.34)	0.819	85/663	0.95 (0.63–1.44)	0.812
University or more	44/156	0.47 (0.34–0.67)	< 0.001	53/156	0.56 (0.40–0.77)	< 0.001	21/156	0.64 (0.38–1.07)	0.086	21/156	0.92 (0.53–1.60)	0.765
Smoking status (pack-years)												
Never smoker	157/493	Reference	–	176/493	Reference	–	67/493	Reference	–	51/493	Reference	–
≤ 23	122/288	1.48 (1.17–1.88)	0.001	132/288	1.44 (1.15–1.81)	0.001	62/288	1.66 (1.18–2.35)	0.004	41/288	1.51 (1.00–2.28)	0.049
> 23	149/277	2.16 (1.73–2.71)	< 0.001	155/277	2.00 (1.61–2.48)	< 0.001	67/277	1.99 (1.42–2.80)	< 0.001	44/277	1.85 (1.23–2.77)	0.003
Alcohol drinking												
Nondrinker	236/628	Reference	–	257/628	Reference	–	109/628	Reference	–	74/628	Reference	–
≤ 1 drink per day	110/251	1.21 (0.97–1.52)	0.099	119/251	1.21 (0.97–1.50)	0.092	53/251	1.28 (0.92–1.78)	0.139	37/251	1.30 (0.88–1.93)	0.192
> 1 drink per day	85/184	1.26 (0.98–1.61)	0.070	90/184	1.22 (0.96–1.55)	0.109	34/184	1.09 (0.74–1.60)	0.666	26/184	1.22 (0.78–1.91)	0.383
Clinical stage												
II	38/178	Reference	–	43/178	Reference	–	16/178	Reference	–	16/178	Reference	–
III	229/601	1.96 (1.39–2.76)	< 0.001	249/601	1.92 (1.39–2.66)	< 0.001	104/601	2.07 (1.22–3.50)	0.007	76/601	1.53 (0.89–2.63)	0.121
IV	164/284	3.63 (2.55–5.17)	< 0.001	174/284	3.46 (2.48–4.84)	< 0.001	76/284	3.47 (2.02–5.95)	< 0.001	45/284	2.21 (1.25–3.90)	0.007

Table S2 Continued

Characteristics	Overall survival			Progression-free survival			Distant metastasis-free survival			Recurrence-free survival		
	n./N.	HR (95%CI)	*P*	*n./N.*	HR (95%CI)	*P*	*n./N.*	HR (95%CI)	*P*	*n./N.*	HR (95%CI)	*P*
T stage												
T_{1-2}	91/297	Reference	–	98/297	Reference	–	38/297	Reference	–	30/297	Reference	–
T_{3-4}	340/766	1.55 (1.23–1.95)	< 0.001	368/766	1.59 (1.27–1.98)	< 0.001	158/766	1.73 (1.21–2.47)	0.003	107/766	1.47 (0.98–2.20)	0.064
N stage												
N_{0-1}	242/670	Reference	–	269/670	Reference	–	105/670	Reference	–	87/670	Reference	–
N_{2-3}	189/393	1.47 (1.21–1.78)	< 0.001	197/393	1.40 (1.16–1.68)	< 0.001	91/393	1.63 (1.23–2.17)	0.001	50/393	1.11 (0.78–1.57)	0.562
Radiotherapy technology												
2D-RT	372/869	Reference	–	396/869	Reference	–	160/869	Reference	–	114/869	Reference	–
3D-CRT	3/14	0.41 (0.13–1.28)	0.125	3/14	0.38 (0.12–1.17)	0.092	2/14	0.72 (0.18–2.91)	0.648	0/14	0 (0-Inf)	0.994
IMRT	51/173	0.58 (0.43–0.77)	< 0.001	61/173	0.67 (0.52–0.88)	0.004	31/173	0.94 (0.64–1.38)	0.757	19/173	0.76 (0.47–1.23)	0.262
Induced chemotherapy												
No	198/548	Reference	–	216/548	Reference	–	80/548	Reference	–	71/548	Reference	–
Yes	233/515	1.41 (1.17–1.71)	< 0.001	250/515	1.40 (1.16–1.67)	< 0.001	116/515	1.67 (1.26–2.23)	< 0.001	66/515	1.10 (0.78–1.53)	0.595
Concurrent chemotherapy												
No	274/621	Reference	–	293/621	Reference	–	127/621	Reference	–	89/621	Reference	–
Yes	157/442	0.70 (0.58–0.86)	< 0.001	173/442	0.74 (0.61–0.89)	0.002	69/442	0.73 (0.54–0.97)	0.032	48/442	0.69 (0.48–0.97)	0.036
Adjuvant chemotherapy												
No	409/1,017	Reference	–	443/1,017	Reference	–	185/1,017	Reference	–	133/1,017	Reference	–
Yes	22/46	1.32 (0.86–2.02)	0.209	23/46	1.26 (0.83–1.92)	0.280	11/46	1.44 (0.78–2.64)	0.244	4/46	0.70 (0.26–1.89)	0.479
EBV DNA (copies/10^6 globin)												
≤ 392	269/752	Reference	–	294/752	Reference	–	115/752	Reference	–	90/752	Reference	–
392–581	49/104	1.49 (1.10–2.02)	0.010	54/104	1.49 (1.12–1.99)	0.007	20/104	1.37 (0.85–2.20)	0.193	12/104	1.10 (0.60–2.01)	0.759
581–918	51/103	1.64 (1.22–2.21)	0.001	54/103	1.60 (1.20–2.14)	0.001	27/103	1.98 (1.30–3.01)	0.001	17/103	1.65 (0.98–2.78)	0.057
> 918	62/104	2.04 (1.55–2.69)	< 0.001	64/104	2.01 (1.53–2.63)	< 0.001	34/104	2.57 (1.75–3.79)	< 0.001	18/104	1.84 (1.11–3.06)	0.018

Table S2 Continued

Characteristics	Overall survival			Progression-free survival			Distant metastasis-free survival			Recurrence-free survival		
	n./*N*.	HR (95%CI)	*P*	*n*./*N*.	HR (95%CI)	*P*	*n*./*N*.	HR (95%CI)	*P*	*n*./*N*.	HR (95%CI)	*P*
History of hypertension												
No	397/994	Reference	–	431/994	Reference	–	188/994	Reference	–	132/994	Reference	–
Yes	34/69	1.34 (0.95–1.91)	0.100	35/69	1.20 (0.85–1.70)	0.290	8/69	0.60 (0.30–1.22)	0.158	5/69	0.55 (0.22–1.34)	0.187
History of diabetes												
No	413/1,031	Reference	–	448/1,031	Reference	–	189/1,031	Reference	–	134/1,031	Reference	–
Yes	18/32	1.56 (0.97–2.50)	0.066	18/32	1.36 (0.85–2.17)	0.205	7/32	1.22 (0.58–2.60)	0.599	3/32	0.75 (0.24–2.34)	0.616
History of heart disease												
No	428/1,055	Reference	–	463/1,055	Reference	–	195/1,055	Reference	–	136/1,055	Reference	–
Yes	3/8	0.95 (0.31–2.97)	0.934	3/8	0.84 (0.27–2.62)	0.765	1/8	0.66 (0.09–4.69)	0.675	1/8	0.99 (0.14–7.06)	0.990

n: the number of events; *N*: the total number of patients in each group.

Table S3 Multivariate Cox regression analysis of all patients

Characteristics	Overall survival			Progression-free survival			Distant metastasis-free survival			Recurrence-free survival		
	n./*N*.	HR (95%CI)	*P*	*n*./*N*.	HR (95%CI)	*P*	*n*./*N*.	HR (95%CI)	*P*	*n*./*N*.	HR (95%CI)	*P*
Age												
≤ 45	176/565	Reference	–	201/565	Reference	–	95/565	Reference	–	72/565	Reference	–
> 45	255/498	1.50 (1.22–1.86)	< 0.001	265/498	1.36 (1.11–1.66)	0.003	101/498	1.11 (0.82–1.5)	0.505	65/498	0.89 (0.62–1.28)	0.532
Gender												
Male	347/775	Reference	–	371/775	Reference	–	164/775	Reference	–	109/775	Reference	–
Female	84/288	0.63 (0.45–0.87)	0.005	95/288	0.66 (0.49–0.90)	0.009	32/288	0.60 (0.37–0.97)	0.038	28/288	0.83 (0.48–1.44)	0.509
Education												
Primary school or less	119/244	Reference	–	125/244	Reference	–	48/244	–	–	31/244	–	–
High school	268/663	0.79 (0.62–1.00)	0.050	288/663	0.80 (0.64–1.01)	0.062	127/663	–	–	85/663	–	–
University or more	44/156	0.64 (0.44–0.93)	0.018	53/156	0.72 (0.51–1.02)	0.067	21/156	–	–	21/156	–	–

Table S3 Continued

Characteristics	Overall survival			Progression-free survival			Distant metastasis-free survival			Recurrence-free survival		
	n./N.	HR (95%CI)	*P*	*n./N.*	HR (95%CI)	*P*	*n./N.*	HR (95%CI)	*P*	*n./N.*	HR (95%CI)	*P*
Smoking status (pack-years)												
Never smoker	157/493	Reference	–	176/493	Reference	–	67/493	Reference	–	51/493	Reference	–
≤ 23	122/288	1.06 (0.79–1.41)	0.698	132/288	1.05 (0.80–1.39)	0.706	62/288	1.14 (0.75–1.72)	0.542	41/288	1.30 (0.78–2.17)	0.312
> 23	149/277	1.25 (0.93–1.67)	0.137	155/277	1.25 (0.94–1.66)	0.119	67/277	1.26 (0.82–1.93)	0.297	44/277	1.57 (0.92–2.67)	0.097
Clinical stage												
II	38/178	Reference	–	43/178	Reference	–	16/178	Reference	–	16/178	Reference	–
III	229/601	2.10 (1.45–3.05)	< 0.001	249/601	2.03 (1.43–2.89)	< 0.001	104/601	2.18 (1.23–3.86)	0.008	76/601	1.86 (1.03–3.35)	0.040
IV	164/284	3.30 (2.20–4.96)	< 0.001	174/284	3.22 (2.19–4.72)	< 0.001	76/284	2.86 (1.54–5.30)	0.001	45/284	2.40 (1.23–4.67)	0.010
Radiotherapy technology												
2D-RT	372/869	Reference	–	396/869	Reference	–	160/869	Reference	–	114/869	Reference	–
3D-CRT	3/14	0.74 (0.24–2.34)	0.610	3/14	0.62 (0.20–1.96)	0.421	2/14	1.18 (0.29–4.88)	0.817	0/14	0 (0-Inf)	0.994
IMRT	51/173	0.76 (0.55–1.05)	0.096	61/173	0.88 (0.65–1.19)	0.399	31/173	1.22 (0.79–1.87)	0.373	19/173	0.96 (0.56–1.64)	0.881
Induced chemotherapy												
No	198/548	Reference	–	216/548	Reference	–	80/548	Reference	–	71/548	Reference	–
Yes	233/515	0.94 (0.76–1.17)	0.589	250/515	0.94 (0.77–1.16)	0.587	116/515	1.14 (0.83–1.58)	0.415	66/515	0.78 (0.53–1.15)	0.216
Concurrent chemotherapy												
No	274/621	Reference	–	293/621	Reference	–	127/621	Reference	–	89/621	Reference	–
Yes	157/442	0.69 (0.55–0.86)	0.001	173/442	0.69 (0.56–0.86)	0.001	69/442	0.57 (0.40–0.81)	0.002	48/442	0.65 (0.44–0.98)	0.038
Adjuvant chemotherapy												
No	409/1,017	Reference	–	443/1,017	Reference	–	185/1,017	Reference	–	133/1,017	Reference	–
Yes	22/46	1.53 (0.98–2.38)	0.063	23/46	1.41 (0.91–2.18)	0.122	11/46	1.62 (0.86–3.07)	0.138	4/46	0.69 (0.25–1.90)	0.472
EBV DNA (copies/10^6 globin)												
≤ 392	269/752	Reference	–	294/752	Reference	–	115/752	Reference	–	90/752	Reference	–
392–581	49/104	1.50 (1.10–2.05)	0.010	54/104	1.50 (1.12–2.01)	0.007	20/104	1.31 (0.81–2.14)	0.272	12/104	1.05 (0.56–1.96)	0.889
581–918	51/103	1.52 (1.12–2.07)	0.007	54/103	1.46 (1.08–1.96)	0.013	27/103	1.76 (1.15–2.69)	0.009	17/103	1.61 (0.95–2.72)	0.077
> 918	62/104	1.85 (1.40–2.46)	< 0.001	64/104	1.85 (1.41–2.44)	< 0.001	34/104	2.37 (1.61–3.51)	< 0.001	18/104	1.70 (1.01–2.87)	0.047

n: the number of events; *N*: the total number of patients in each group.

Table S4 Relationship between peripheral blood cell Epstein-Barr virus DNA loads and other factors

Characteristics	Low group (≤ 392 copies)	High group (> 392 copies)	OR (95%CI)[a]	*P*[a]	OR (95%CI)[b]	*P*[b]
Age						
≤ 45	400 (70.8%)	165 (29.2%)	Reference	–	Reference	–
> 45	352 (70.7%)	146 (29.3%)	1.00 (0.77–1.31)	0.968	0.98 (0.73–1.32)	0.888
Gender						
Male	545 (70.3%)	230 (29.7%)	Reference	–	Reference	–
Female	207 (71.9%)	81 (28.1%)	0.93 (0.69–1.25)	0.621	1.19 (0.77–1.83)	0.426
BMI						
18.5–24.0	392 (68.1%)	184 (31.9%)	Reference	–	Reference	–
> 24.0	230 (76.4%)	71 (23.6%)	0.66 (0.48–0.90)	0.010	0.70 (0.50–0.97)	0.033
< 18.5	69 (69.0%)	31 (31.0%)	0.96 (0.61–1.51)	0.852	1.00 (0.62–1.60)	0.994
Education						
Primary school or less	166 (68.0%)	78 (32.0%)	Reference	–		
High school	476 (71.8%)	187 (28.2%)	0.84 (0.61–1.15)	0.270		
University or more	110 (70.5%)	46 (29.5%)	0.89 (0.57–1.38)	0.601		
Smoking status (pack-years)				0.407		
Never smoker	357 (72.4%)	136 (27.6%)	Reference	–	Reference	–
≤ 23	202 (70.1%)	86 (29.9%)	1.12 (0.81–1.54)	0.497	1.13 (0.74–1.73)	0.571
> 23	188 (67.9%)	89 (32.1%)	1.24 (0.90–1.71)	0.184	1.21 (0.78–1.87)	0.392
Alcohol drinking				0.293		
Nondrinker	455 (72.5%)	173 (27.5%)	Reference	–		
≤ 1 drink per day	174 (69.3%)	77 (30.7%)	1.16 (0.84–1.60)	0.353		
> 1 drink per day	123 (66.8%)	61 (33.2%)	1.30 (0.92–1.86)	0.141		
Clinical stage						
II	148 (83.1%)	30 (16.9%)	Reference	–	Reference	–
III	428 (71.2%)	173 (28.8%)	1.99 (1.30–3.07)	0.002	1.71 (1.05–2.79)	0.032
IV	176 (62.0%)	108 (38.0%)	3.03 (1.91–4.79)	< 0.001	2.45 (1.41–4.24)	0.001
T stage						
T_{1-2}	225 (75.8%)	72 (24.2%)	Reference	–		
T_{3-4}	527 (68.8%)	239 (31.2%)	1.42 (1.04–1.93)	0.026		
N stage						
N_{0-1}	518 (77.3%)	152 (22.7%)	Reference	–		
N_{2-3}	234 (59.5%)	159 (40.5%)	2.32 (1.77–3.04)	< 0.001		

Table S4 Continued

Characteristics	Low group (≤ 392 copies)	High group (> 392 copies)	OR (95%CI)[a]	*P*[a]	OR (95%CI)[b]	*P*[b]
Radiotherapy technology				0.799		
2D-RT	614 (70.7%)	255 (29.3%)	Reference	–	Reference	–
3D-CRT	11 (78.6%)	3 (21.4%)	0.66 (0.18–2.37)	0.521	0.70 (0.19–2.63)	0.603
IMRT	122 (70.5%)	51 (29.5%)	1.00 (0.70–1.44)	0.971	0.96 (0.64–1.45)	0.864
Induced chemotherapy						
No	415 (75.7%)	133 (24.3%)	Reference	–	Reference	–
Yes	337 (65.4%)	178 (34.6%)	1.63 (1.25–2.13)	< 0.001	1.25 (0.92–1.72)	0.159
Concurrent chemotherapy						
No	445 (71.7%)	176 (28.3%)	Reference	–	Reference	–
Yes	307 (69.5%)	135 (30.5%)	1.11 (0.85–1.45)	0.437	1.07 (0.77–1.49)	0.665
Adjuvant chemotherapy						
No	722 (71.0%)	295 (29.0%)	Reference	–	Reference	–
Yes	30 (65.2%)	16 (34.8%)	1.31 (0.70–2.43)	0.401	1.20 (0.61–2.36)	0.595
History of hypertension						
No	705 (70.9%)	289 (29.1%)	Reference	–		
Yes	47 (68.1%)	22 (31.9%)	1.14 (0.68–1.93)	0.620		
History of diabetes						
No	730 (70.8%)	301 (29.2%)	Reference	–		
Yes	22 (68.8%)	10 (31.3%)	1.10 (0.52–2.36)	0.801		
History of heart disease						
No	745 (70.6%)	310 (29.4%)	Reference	–		
Yes	7 (87.5%)	1 (12.5%)	0.34 (0.04–2.80)	0.318		

a: the results of univariate analysis. b: the results of multivariate analysis.

Table S5 Multivariate Cox regression analysis in the clinical stage II subgroup

Characteristics	Overall survival			Progression-free survival			Distant metastasis-free survival			Recurrence-free survival		
	n./N.	HR (95%CI)	*P*	*n./N.*	HR (95%CI)	*P*	*n./N.*	HR (95%CI)	*P*	*n./N.*	HR (95%CI)	*P*
Age												
≤ 45	15/100	Reference	–	18/100	Reference	–	8/100	Reference	–	7/100	Reference	–
> 45	23/78	2.58 (1.15–5.77)	0.021	25/78	2.11 (1.02–4.36)	0.043	8/78	1.90 (0.58–6.16)	0.287	9/78	1.82 (0.58–5.69)	0.301
Gender												
Male	31/127	Reference	–	34/127	Reference	–	12/127	Reference	–	13/127	Reference	–
Female	7/51	0.81 (0.29–2.25)	0.693	9/51	0.94 (0.37–2.37)	0.898	4/51	2.86 (0.45–18.11)	0.263	3/51	0.77 (0.17–3.44)	0.729
Education												
Primary school or less	10/31	Reference	–	10/31	Reference	–	4/31	–	–	3/31	–	–
High school	23/110	0.72 (0.32–1.64)	0.438	26/110	0.82 (0.37–1.83)	0.633	12/110	–	–	9/110	–	–
University or more	5/37	0.65 (0.21–2.01)	0.452	7/37	0.93 (0.34–2.59)	0.894	0/37	–	–	4/37	–	–
Smoking status (pack-years)												
Never smoker	19/87	Reference	–	22/87			8/87			10/87		
≤ 23	10/60	1.67 (0.68–4.06)	0.261	11/60	1.65 (0.71–3.83)	0.242	2/60	5.22 (0.88–30.94)	0.069	3/60	1.36 (0.36–5.07)	0.650
> 23	7/16	1.74 (0.60–5.06)	0.312	8/16	1.78 (0.66–4.83)	0.257	5/16	4.15 (0.54–31.74)	0.170	3/16	1.33 (0.28–6.46)	0.721
Radiotherapy technology												
2D-RT	33/151	Reference	–	38/151	Reference	–	14/151	Reference	–	13/151	Reference	–
3D-CRT	0/5	0 (0-Inf)	0.996	0/5	0 (0-Inf)	0.996	0/5	0 (0-Inf)	0.998	0/5	0 (0-Inf)	0.998
IMRT	4/21	1.23 (0.40–3.76)	0.717	6/25	1.03 (0.34–3.07)	0.965	1/21	0.89 (0.11–7.47)	0.912	2/21	1.33 (0.27–6.55)	0.723
Induced chemotherapy												
No	34/165	Reference	–	39/165	Reference	–	14/165	Reference	–	15/165	Reference	–
Yes	4/13	2.28 (0.67–7.80)	0.188	4/13	1.60 (0.49–5.22)	0.437	2/13	1.59 (0.31–8.28)	0.579	1/13	1.25 (0.14–11.32)	0.842
Concurrent chemotherapy												
No	33/147	Reference	–	38/147	Reference	–	15/147	Reference	–	12/147	Reference	–
Yes	5/31	0.62 (0.23–1.68)	0.350	5/31	0.54 (0.20–1.42)	0.209	1/31	0.31 (0.04–2.47)	0.267	4/31	1.42 (0.42–4.75)	0.569

Table S5 Continued

Characteristics	Overall survival			Progression-free survival			Distant metastasis-free survival			Recurrence-free survival		
	n./N.	HR (95%CI)	*P*	*n./N.*	HR (95%CI)	*P*	*n./N.*	HR (95%CI)	*P*	*n./N.*	HR (95%CI)	*P*
Adjuvant chemotherapy												
No	38/178	–	–	43/178	–	–	16/178	–	–	16/178	–	–
Yes	0/0	–	–	0/0	–	–	0/0	–	–	0/0	–	–
EBV DNA (copies/10^6 globin)												
≤ 392	27/148	Reference	–	29/148	Reference	–	9/148	Reference	–	11/148	Reference	–
> 392	11/30	2.72 (1.29–5.73)	0.008	14/30	3.38 (1.72–6.65)	< 0.001	7/30	4.96 (1.71–14.34)	0.003	5/30	3.31 (1.06–10.31)	0.039

n.: the number of events; *N.*: the total number of patients in each group.

Table S6 Multivariate Cox regression analysis in the clinical stage III subgroup

Characteristics	Overall survival			Progression-free survival			Distant metastasis-free survival			Recurrence-free survival		
	n./N.	HR (95%CI)	*P*	*n./N.*	HR (95%CI)	*P*	*n./N.*	HR (95%CI)	*P*	*n./N.*	HR (95%CI)	*P*
Age												
≤ 45	101/326	Reference	–	116/326	Reference	–	54/326	Reference	–	46/326	Reference	–
> 45	128/275	1.40 (1.05–01.86)	0.023	133/275	1.22 (0.93–1.60)	0.161	50/275	1.13 (0.75–1.70)	0.562	30/275	0.66 (0.41–1.08)	0.099
Gender												
Male	171/406	Reference	–	182/406	Reference	–	86/406	Reference	–	54/406	Reference	–
Female	58/195	0.58 (0.38–0.89)	0.013	67/195	0.68 (0.45–1.01)	0.058	18/195	0.48 (0.25–0.91)	0.026	22/195	0.99 (0.49–2.00)	0.980
Education												
Primary school or less	73/149	Reference	–	78/149	Reference	–	26/149	–	–	18/149	–	–
High school	130/362	0.73 (0.53–1.00)	0.050	142/362	0.74 (0.55–1.01)	0.057	68/362	–	–	47/362	–	–
University or more	26/90	0.63 (0.39–1.04)	0.070	29/90	0.63 (0.39–1.01)	0.055	10/90	–	–	11/90	–	–

Table S6 Continued

Characteristics	Overall survival			Progression-free survival			Distant metastasis-free survival			Recurrence-free survival		
	n./N.	HR (95%CI)	*P*	*n./N.*	HR (95%CI)	*P*	*n./N.*	HR (95%CI)	*P*	*n./N.*	HR (95%CI)	*P*
Smoking status (pack-years)												
Never smoker	94/304	Reference	–	106/304	Reference	–	37/304	Reference	–	34/304	Reference	–
≤ 23	58/142	1.14 (0.76–1.72)	0.512	63/142	1.24 (0.84–1.83)	0.281	36/142	1.48 (0.85–2.58)	0.165	20/142	1.53 (0.75–3.12)	0.238
> 23	76/153	1.28 (0.85–1.91)	0.237	79/153	1.33 (0.90–1.98)	0.150	31/153	1.23 (0.68–2.21)	0.495	22/153	1.85 (0.89–3.82)	0.099
Radiotherapy technology												
2D-RT	201/488	Reference	–	215/488	Reference	–	84/488	Reference	–	65/488	Reference	–
3D-CRT	1/5	0.47 (0.07–3.46)	0.463	1/5	0.42 (0.06–3.04)	0.388	1/5	1.58 (0.21–11.91)	0.656	0/5	0 (0-Inf)	0.994
IMRT	26/105	0.59 (0.38–0.93)	0.024	31/105	0.69 (0.45–1.06)	0.088	19/105	1.32 (0.74–2.36)	0.347	10/105	0.86 (0.41–1.81)	0.685
Induced chemotherapy												
No	117/310	Reference	–	129/310	Reference	–	47/310	Reference	–	44/310	Reference	–
Yes	112/291	1.13 (0.87–1.48)	0.363	120/291	1.09 (0.84–1.41)	0.500	57/291	1.26 (0.85–1.87)	0.245	32/291	0.76 (0.47–1.2)	0.239
Concurrent chemotherapy												
No	138/324	Reference	–	149/324	Reference	–	65/324	Reference	–	50/324	Reference	–
Yes	91/277	0.72 (0.53–0.97)	0.030	100/277	0.73 (0.55–0.98)	0.038	39/277	0.52 (0.32–0.84)	0.008	26/277	0.55 (0.32–0.95)	0.031
Adjuvant chemotherapy												
No	217/571	Reference	–	236/571	Reference	–	97/571	Reference	–	75/571	Reference	–
Yes	12/30	1.52 (0.83–2.79)	0.176	13/30	1.38 (0.77–2.48)	0.274	7/30	1.97 (0.87–4.45)	0.104	1/30	0.30 (0.04–2.20)	0.236
EBV DNA (copies/10^6 globin)												
≤ 392	153/428	Reference	–	169/428	Reference	–	68/428	Reference	–	56/428	Reference	–
> 392	76/173	1.38 (1.04–1.82)	0.025	80/173	1.27 (0.97–1.67)	0.081	36/173	1.42 (0.94–2.14)	0.093	20/173	0.97 (0.58–1.63)	0.919

n.: the number of events; *N.*: the total number of patients in each group.

Table S7 Multivariate Cox regression analysis in the clinical stage IV subgroup

Characteristics	Overall survival			Progression-free survival			Distant metastasis-free survival			Recurrence-free survival		
	n./N.	HR (95%CI)	*P*	*n./N.*	HR (95%CI)	*P*	*n./N.*	HR (95%CI)	*P*	*n./N.*	HR (95%CI)	*P*
Age												
≤ 45	60/139	Reference	–	67/139	Reference	–	33/139	Reference	–	19/139	Reference	–
> 45	104/145	1.55 (1.08–2.23)	0.017	107/145	1.43 (1.01–2.02)	0.043	43/145	1.02 (0.60–1.72)	0.940	26/145	1.06 (0.54–2.09)	0.863
Gender												
Male	145/242	Reference	–	155/242	Reference	–	66/242	Reference	–	42/242	Reference	–
Female	19/42	0.80 (0.43–1.49)	0.480	19/42	0.61 (0.34–1.12)	0.113	10/42	0.75 (0.33–1.68)	0.484	3/42	0.51 (0.13–2.00)	0.336
Education												
Primary school or less	36/64	Reference	–	37/64	Reference	–	18/64	–	–	10/64	–	–
High school	115/191	0.92 (0.61–1.41)	0.712	120/191	0.91 (0.61–1.37)	0.652	47/191	–	–	29/191	–	–
University or more	13/29	0.69 (0.35–1.36)	0.278	17/29	0.90 (0.48–1.67)	0.734	11/29	–	–	6/29	–	–
Smoking status (pack-years)												
Never smoker	48/91	Reference	–	52/91	Reference	–	25/91	Reference	–	10/91	Reference	–
≤ 23	49/95	0.91 (0.56–1.45)	0.680	53/95	0.77 (0.49–1.20)	0.242	18/95	0.56 (0.28–1.12)	0.099	15/95	1.08 (0.44–2.69)	0.862
> 23	66/97	1.15 (0.72–1.84)	0.567	68/97	1.02 (0.65–1.59)	0.935	33/97	1.06 (0.55–2.06)	0.857	19/97	1.32 (0.52–3.35)	0.554
T stage												
T_{1-2}	10/20	Reference	–	11/20	Reference	–	8/20	Reference	–	4/20	Reference	–
T_{3-4}	154/264	1.51 (0.72–3.14)	0.272	163/264	1.29 (0.64–2.60)	0.480	68/264	1.00 (0.41–2.47)	0.999	41/264	0.97 (0.27–3.45)	0.960
N stage												
N_{0-1}	90/162	Reference	–	97/162	Reference	–	37/162	Reference	–	25/162	Reference	–
N_{2-3}	74/122	1.33 (0.94–1.87)	0.105	77/122	1.20 (0.86–1.68)	0.275	39/122	1.46 (0.88–2.41)	0.144	20/122	1.16 (0.59–2.26)	0.666
Radiotherapy technology												
2D-RT	138/230	Reference	–	143/230	Reference	–	62/230	Reference	–	36/230	Reference	–
3D-CRT	2/4	1.12 (0.26–4.78)	0.879	2/4	1.00 (0.24–4.23)	0.999	1/4	0.98 (0.13–7.56)	0.983	0/4	0 (0-Inf)	0.997
IMRT	21/47	1.00 (0.60–1.67)	0.993	26/47	1.25 (0.78–2.01)	0.348	11/47	1.12 (0.55–2.29)	0.755	7/47	1.34 (0.54–3.35)	0.532

Table S7 Continued

Characteristics	Overall survival			Progression-free survival			Distant metastasis-free survival			Recurrence-free survival		
	n./*N.*	HR (95%CI)	*P*	*n.*/*N.*	HR (95%CI)	*P*	*n.*/*N.*	HR (95%CI)	*P*	*n.*/*N.*	HR (95%CI)	*P*
Induced chemotherapy												
No	47/73	Reference	–	48/73	Reference	–	19/73	Reference	–	12/73	Reference	–
Yes	117/211	0.65 (0.45–0.93)	0.020	126/211	0.70 (0.49–1.00)	0.052	57/211	0.87 (0.50–1.51)	0.613	33/211	0.88 (0.42–1.85)	0.728
Concurrent chemotherapy												
No	103/150	Reference	–	106/150	Reference	–	47/150	Reference	–	27/150	Reference	–
Yes	61/134	0.66 (0.45–0.96)	0.031	68/134	0.67 (0.46–0.96)	0.030	29/134	0.66 (0.38–1.15)	0.145	18/134	0.74 (0.36–1.52)	0.412
Adjuvant chemotherapy												
No	154/268	Reference	–	164/268	Reference	–	72/268	Reference	–	42/268	Reference	–
Yes	10/16	1.37 (0.70–2.69)	0.364	10/16	1.23 (0.63–2.41)	0.543	4/16	1.20 (0.42–3.45)	0.733	3/16	1.26 (0.36–4.35)	0.720
EBV DNA (copies/10^6 globin)												
≤ 392	89/176	Reference	–	96/176	Reference	–	38/176	Reference	–	23/176	Reference	–
> 392	75/108	1.92 (1.40–2.63)	< 0.001	78/108	1.93 (1.41–2.63)	< 0.001	38/108	2.07 (1.30–3.29)	0.002	22/108	2.15 (1.16–3.98)	0.015

n.: the number of events; *N.*: the total number of patients in each group.

Table S8 The scoring system of the nomogram for overall survival and progression-free survival

Characteristics	Overall survival score	Progression-free survival score
Age		
≤ 45	0.0	0.0
> 45	59.4	59.8
Gender		
Male	65.4	66.1
Female	0.0	0.0
BMI		
18.5–24.0	0.0	10.6
> 24.0	4.0	0.0
< 18.5	11.7	11.3
Education		
Primary school or less	71.0	67.6
High school	35.6	28.0
University or more	0.0	0.0
Smoking status (pack-years)		
Never smoker	0.0	0.0
≤ 23	20.2	21.2
> 23	37.6	48.9
T stage		
T_{1-2}	0.0	0.0
T_{3-4}	27.6	26.5
N stage		
N_{0-1}	0.0	0.0
N_{2-3}	25.2	16.9
Concurrent chemotherapy		
No	60.7	61.2
Yes	0.0	0.0
EBV DNA (copies/10^6 globin)		
≤ 392	0.0	0.0
392–581	5.1	19.2
581–918	56.7	53.0
> 918	80.1	86.9
LDH		
< 245	0.0	0.0
≥ 245	100.0	100.0
NLR		
≤ 1.33	0.0	0.0
> 1.33	19.8	26.4

Table S9 Multivariate Cox analysis after adjusted plasma Epstein-Barr virus DNA loads in 205 patients

Characteristics	*n./N.*	HR (95%CI)	*P*
Age			
≤ 45	30/111	Reference	–
> 45	48/94	1.15 (0.67–1.96)	0.607
Gender			
Male	65/153	Reference	–
Female	13/52	0.82 (0.37–1.79)	0.612
Smoking status (pack-years)			
Never smoker	27/96	Reference	–
≤ 23	21/55	1.09 (0.55–2.18)	0.802
> 23	30/54	1.34 (0.68–2.65)	0.394
Clinical stage			
II	4/32	Reference	–
III	37/115	2.74 (0.90–8.36)	0.076
IV	37/58	5.32 (1.67–17.00)	0.005
Radiotherapy technology			
2D-RT	64/146	Reference	–
3D-CRT	0/0	–	–
IMRT	13/56	0.47 (0.23–0.96)	0.038
Induced chemotherapy			
No	27/96	Reference	–
Yes	51/109	0.88 (0.49–1.58)	0.672
Concurrent chemotherapy			
No	49/115	Reference	–
Yes	29/90	0.67 (0.37–1.20)	0.179
Adjuvant chemotherapy			
No	75/200	Reference	–
Yes	3/5	2.84 (0.80–10.14)	0.108
PBC EBV DNA loads			
≤ 392 copies/10^6 globin	56/160	Reference	–
> 392 copies/10^6 globin	22/45	1.88 (1.08–3.26)	0.025
Plasma EBV DNA loads			
≤ 128,000 copies/mL	38/128	Reference	–
> 128,000 copies/mL	40/77	1.50 (0.90–2.52)	0.121

n.: the number of events; *N.*: the total number of patients in each group.

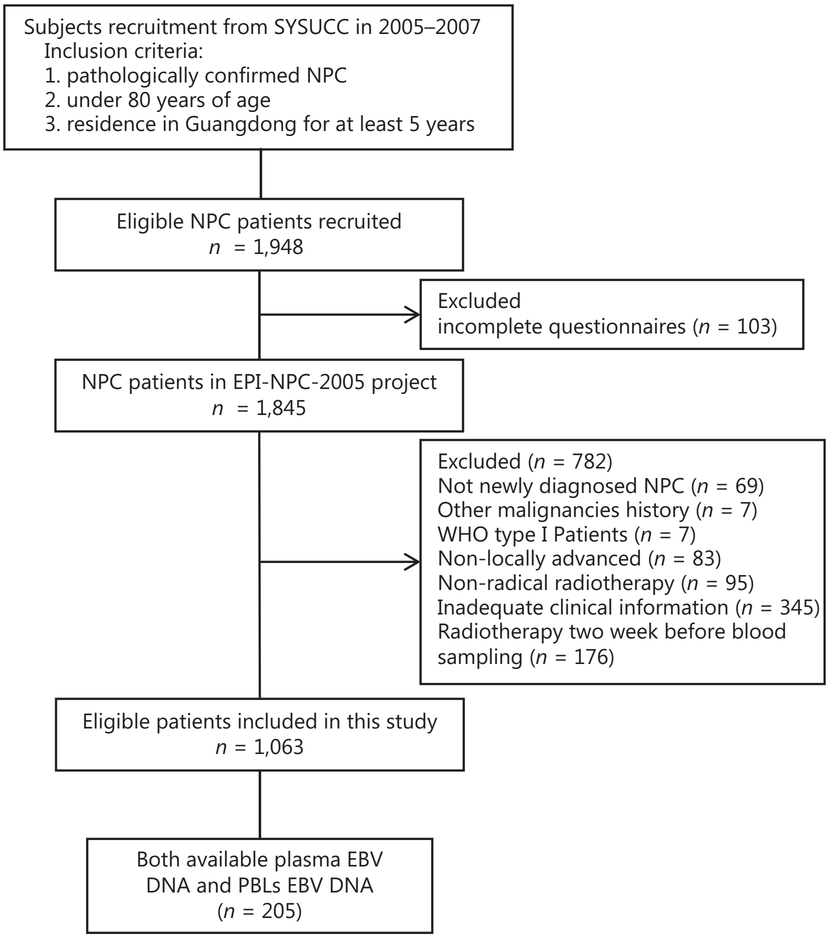

Figure S1 A flow chart of patient inclusion. NPC = nasopharyngeal carcinoma; SYSUCC = Sun Yat-sen University Cancer Center; PBC = peripheral blood cells.

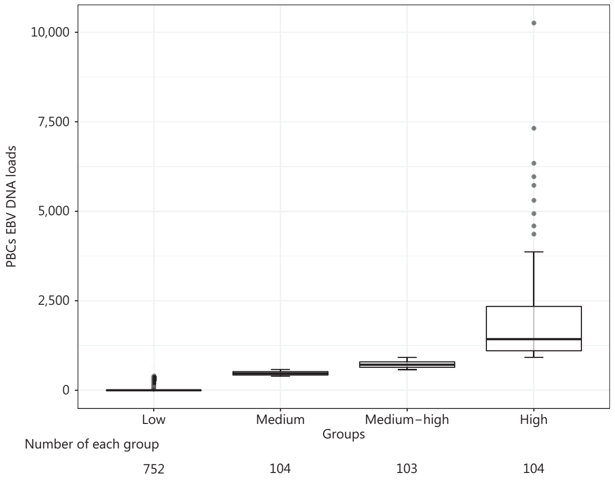

Figure S2 A box plot for peripheral blood cell Epstein-Barr virus DNA loads in the low, medium, medium-high, and high groups.

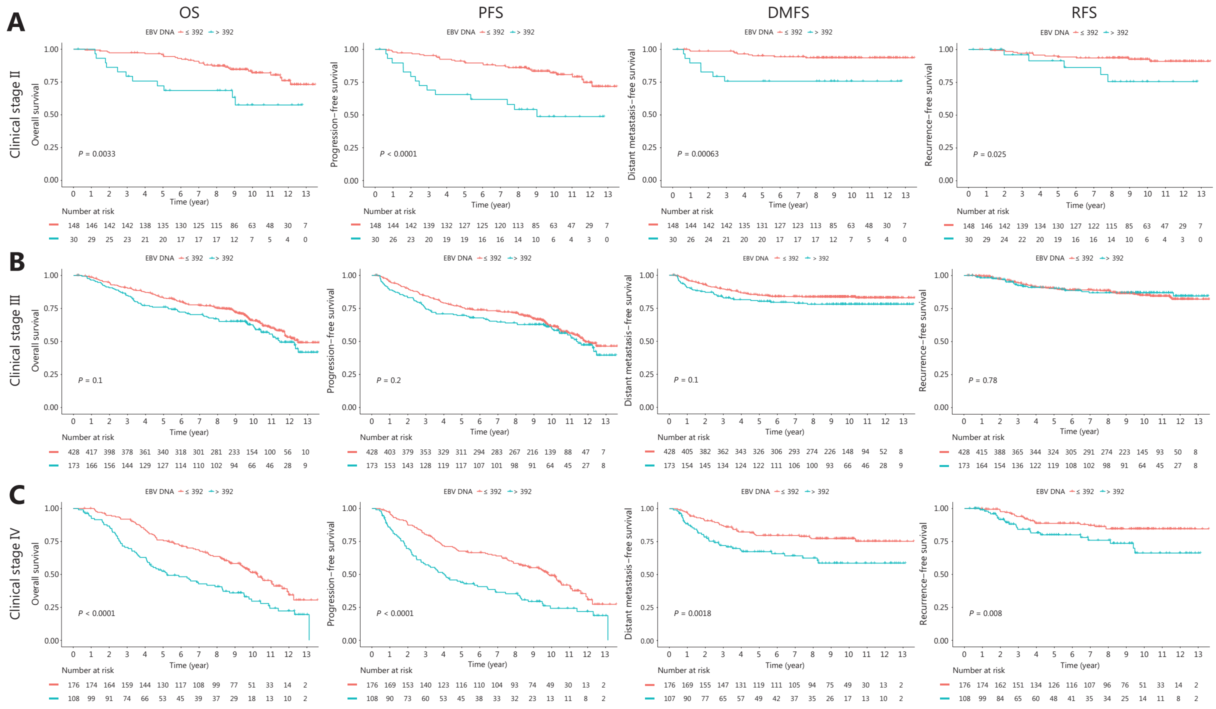

Figure S3 Kaplan-Meier survival curves of Epstein-Barr virus DNA in peripheral blood cells for overall survival, progression-free survival, and distant metastasis-free survival, recurrence-free survival in subgroups of (A) clinical stage II, (B) clinical stage III, and (C) clinical stage IV.

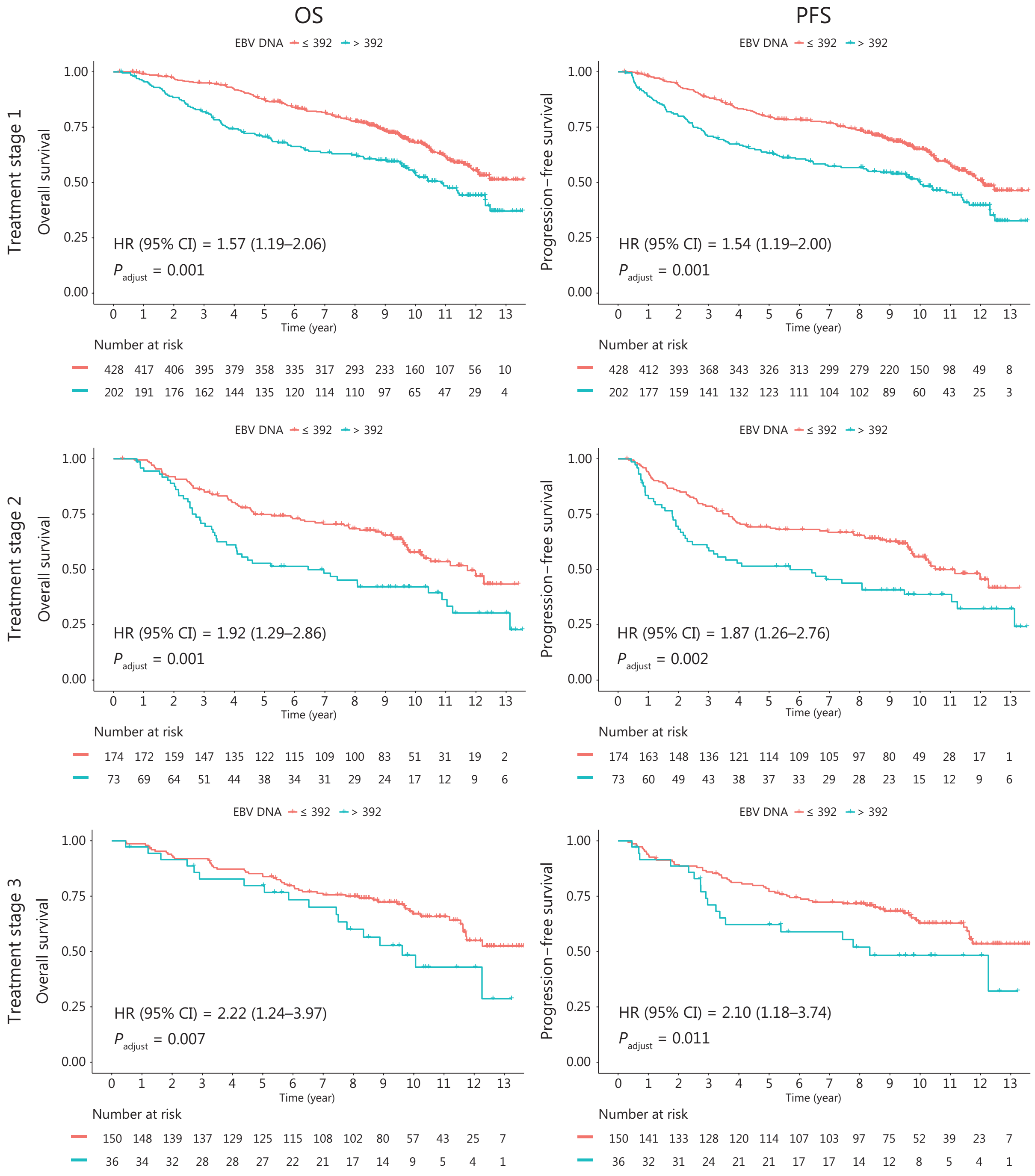

Figure S4 Kaplan-Meier survival curves of Epstein-Barr virus DNA in peripheral blood cells for overall survival and progression-free survival in different treatment stages. The blood samples of patients in treatment stage 1 were collected before any treatment, treatment stage 2 was during induced chemotherapy, and treatment stage 3 was within 2 weeks after the start of radiotherapy. P_{adjust} and hazard ratio (95% confidence interval) were based on the results of multivariable Cox regression that adjusted for age, gender, smoking status, T stage, N stage, radiotherapy technology, chemotherapy (induced chemotherapy, concurrent chemotherapy, adjuvant chemotherapy), and education level.

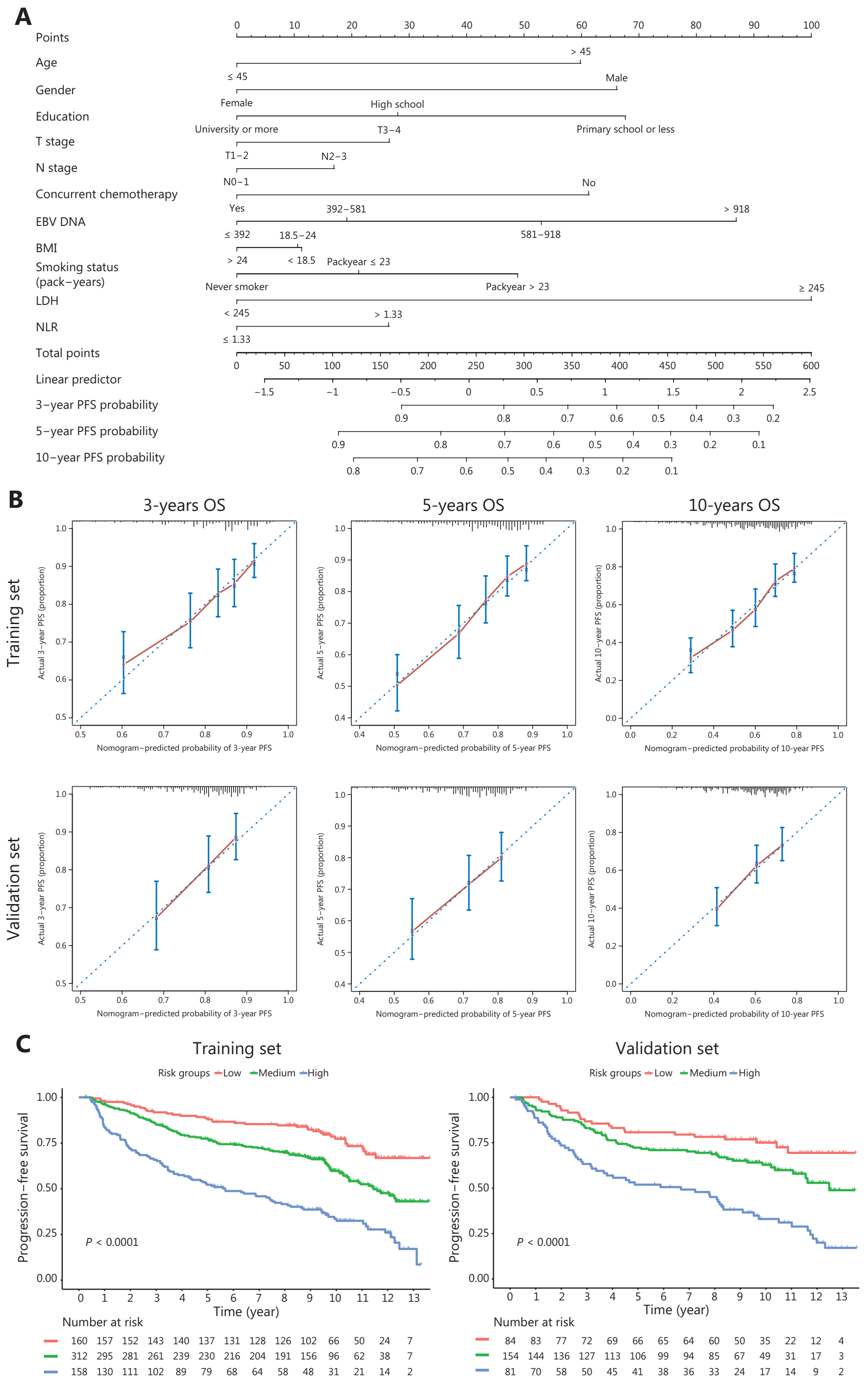

Figure S5 Nomogram (A), including age, gender, education level, T stage, N stage, concurrent chemotherapy, body mass index, smoking status, serum lactate dehydrogenase level, and neutrophil to lymphocyte ratio for 3-, 5-, and 10-year progression-free survival (PFS) for patients with nasopharyngeal carcinoma. The calibration curve (B) of the nomogram for predicting the PFS. The Kaplan-Meier curves (C) for PFS in the training and validation sets.
